# Genes and Genetic Testing in Hereditary Ataxias

**DOI:** 10.3390/genes5030586

**Published:** 2014-07-22

**Authors:** Erin Sandford, Margit Burmeister

**Affiliations:** 1 Molecular & Behavioral Neuroscience Institute, University of Michigan, Ann Arbor, MI 48109, USA; E-Mail: esandfor@umich.edu; 2 Departments of Human Genetics, University of Michigan, Ann Arbor, MI 48109, USA; 3 Department of Computational Medicine & Bioinformatics, University of Michigan, Ann Arbor, MI 48109, USA; 4 Department of Psychiatry, University of Michigan, Ann Arbor, MI 48109, USA

**Keywords:** ataxia, genetics, pathways, testing

## Abstract

Ataxia is a neurological cerebellar disorder characterized by loss of coordination during muscle movements affecting walking, vision, and speech. Genetic ataxias are very heterogeneous, with causative variants reported in over 50 genes, which can be inherited in classical dominant, recessive, X-linked, or mitochondrial fashion. A common mechanism of dominant ataxias is repeat expansions, where increasing lengths of repeated DNA sequences result in non-functional proteins that accumulate in the body causing disease. Greater understanding of all ataxia genes has helped identify several different pathways, such as DNA repair, ubiquitination, and ion transport, which can be used to help further identify new genes and potential treatments. Testing for the most common mutations in these genes is now clinically routine to help with prognosis and treatment decisions, but next generation sequencing will revolutionize how genetic testing will be done. Despite the large number of known ataxia causing genes, however, many individuals with ataxia are unable to obtain a genetic diagnosis, suggesting that more genes need to be discovered. Utilization of next generation sequencing technologies, expression studies, and increased knowledge of ataxia pathways will aid in the identification of new ataxia genes.

## 1. Introduction

Ataxia is a neurological sign that involves a lack of coordinated muscle movement, which impacts walking, speech, and vision. Ataxia can present as an isolated symptom, or present as one of many symptoms of a more complex disease. Acquired ataxias may be temporary or permanent, and can be caused by environmental factors, such as alcohol, trauma, or exposure to toxins, or by other underlying medical conditions such as stroke, infection, tumors, or vitamin deficiencies. However, many ataxias have an underlying genetic cause. Hereditary ataxias are a group of highly heterogeneous diseases, but each usually follows a typical Mendelian dominant, recessive, or X-linked inheritance. The prevalence of hereditary ataxias varies by population and has been estimated at 1–9 per 100,000 people [1,2,3,4]. Many hereditary diseases also present with ataxia as one symptom of a more complex phenotype. This review will focus on disorders classified primarily as ataxia, along with those ataxias that result in other symptoms like intellectual disability, with known genetic association.

Early work on the genetic origins of ataxia began in 1993 with the discovery of a CAG repeat responsible for spinocerebellar ataxia (SCA) type 1 [5]. Continued screening for CAG repeat expansions identified several additional dominant SCAs that are caused by the same mechanism [6,7,8,9,10]. With the advancement of next generation sequencing technology, genome and exome sequencing have become an affordable option for screening for disease genes. Exome sequencing for Mendelian diseases first gained prominence in 2010 with the discovery of the disease gene for Miller syndrome and since then, mutations in several new ataxia genes have been identified utilizing exome sequencing, including *ATP2B3*, *KCND3*, *DNMT1*, *UCHL1*, and *TPP1*, illustrating the utility of the technology [11,12,13,14,15,16,17]. While mutations in many of these new genes were found in only one family (“private” mutations), thus far, mutations in *KCND3* were found in multiple different families on several continents [13,14]. Despite these advances, it is estimated that up to 40% of those with ataxia do not know the genetic cause, illustrating the need to continue research into the identification of ataxia genes in order to provide a diagnosis and potentially a treatment [18].

## 2. Phenotypes of Hereditary Ataxias

Hereditary ataxias exhibit a wide range of phenotypes, in both clinical features and age of onset. Some ataxias are described as “pure cerebellar”, where symptoms are all related to cerebellar control of muscle movement. This can include ataxic gait and movement of body and limbs, along with nystagmus, dysarthia, and hypotonia. Many of these features are easily observed by external examination. Magnetic resonance imaging often provides the clearest explanation for the ataxia through the identification of cerebellar atrophy, but may appear normal in some cases [19,20]. Other ataxias can present with more extensive additional neurological symptoms, such as Parkinsonism, epilepsy, dementia, and neuropathy. Multisystem involvement can include symptoms such as deafness and intellectual disability. These symptoms may be progressive, gradually becoming more severe over time, or non-progressive, where the symptoms are stable.

The age of symptom onset in affected individuals can vary dramatically, both within and across different ataxias, with symptoms present from birth through onset in the 7th and 8th decades of life. Late onset ataxias are more commonly progressive and can result in patients becoming wheelchair bound or even experience a reduced lifespan. Congenital ataxias display symptoms within the first year of life and are often non-progressive, however many congenital ataxias more often present as multisystem diseases. These children may display muscular hypotonia prior to onset of ataxia symptoms, resulting in “floppy baby syndrome”.

A common phenomenon in the dominantly inherited ataxias is anticipation, where the younger generation exhibits symptoms at an earlier age. The rate of anticipation can vary, depending on genetic and environmental factors, but differences in age of onset, up to 20 years, have been reported. Much, but not all, of anticipation can be explained by increasing repeat length of the CAG expansions (see Section 3.1.1). Anticipation can be difficult for clinicians to correctly diagnose, as younger individuals with a family history of ataxia may describe more psychosomatic symptoms in the expectation of developing symptoms later in life.

## 3. Ataxia Genetics

Hereditary ataxias are genetically and phenotypically heterogeneous. Similar phenotypes may be caused by mutations in many different genes, and several genes cause different types of ataxia depending upon the mutation. While many ataxias appear worldwide, such as Friedreich’s ataxia or SCA3, others are more common in one population. Dentatorubral-pallidoluysian atrophy (DRPLA) is most common in Japan and SCA2 is prevalent in Cuba. Other ataxias may be completely restricted to certain populations, such as Cayman ataxia on the Cayman Islands. Knowledge of a patient’s ethnic origin can, therefore, be helpful, along with phenotype and family history. In most newly diagnosed cases with ataxia, screening panels for many ataxia genes is recommended. As ataxia can be misdiagnosed as multiple sclerosis or Parkinson’s, finding a genetic cause often solidifies a diagnosis, not only for an individual, but for the whole family.

### 3.1. Autosomal Dominant

Many dominant ataxias have been classified as SCAs or episodic ataxias (EA). At least 34 different SCAs and seven EAs have been described clinically, with 28 having known associated genetic mutations. Dominant ataxias tend to have an onset later in life and be slowly progressive. SCAs, particularly those caused by repeat expansions, can exhibit a larger range of symptom onset and a faster rate of progression. A detailed review of the clinical characteristics of SCAs was published in 2009 [21]. Individuals with EA experience episodes of ataxia that can range from minutes to hours in duration and are triggered by environmental stimuli such as stress, alcohol, or exercise [22,23,24].

In some cases, the causative gene was identical in several previously reported SCAs, so SCA15, SCA16, and SCA29 all are caused by mutations in *ITPR1* and SCA19 and SCA22 are caused by mutations in *KCND3* [13,14,25,26,27]. Repeat expansion in *CACNA1A* results in SCA6 while single nucleotide variants (SNV), insertions, and deletions result in EA2. Known autosomal dominant (AD) ataxia genes are reported in Table S1.

#### 3.1.1. Repeat Expansions

The most common forms of dominant ataxias are caused by repeat expansion. Short repeats, typically three to six bases long, appear at variable repeat number within many genes. Occasionally these repeat regions become unstable during replication, leading to either deletions of repeats, which rarely causes problems, or to expansion of the number of repeats. Typically within a repeat region, there are instances of non-repeated bases, such as a CAA in a string of CAG. Mutations that convert these imperfections in the repeat region to match the surrounding repeats result in an unstable sequence and increased likelihood of expansion. In ataxias, the number of repeats may increase anywhere from less than 2 to over 100 fold, depending on the gene. The most common repeat expansions are CAG expansions. As CAG encodes glutamine, these are also referred to as a polyglutamine or polyQ repeats, as these repeats form strings of glutamines (Q) in the coding region. There are currently seven known AD ataxias caused by CAG polyglutamine expansions: SCA1, SCA2, SCA3 (also known as Machado Joseph disease or MJD), SCA6, SCA7, SCA17, and DRPLA. In addition, repeat expansions outside the coding region, in introns or the untranslated regions of the gene, also can cause ataxia without causing polyglutamine disease, but rather by interfering with the regulation of the gene: SCA8 (CTG), SCA10 (ATTCT), SCA12 (CAG), SCA31 (TGGAA), and SCA36 (GGCCTG).

The most common SCAs reported are SCA1, SCA2, SCA3, SCA6, and SCA7. Rates for each vary by population; the National Ataxia Foundation reports that SCA6 is responsible for up the 30% of dominant ataxia cases in Japan, but only 15% in the U.S. and 2% in Italy. Together these five SCA make up about 60% of the reported dominant cases of ataxia [21]. With the high frequency of these SCAs, it is not surprising that they were the first genetic mutations responsible for SCA that were identified.

Age of onset is highly variable with repeat expansion disorders, ranging from early childhood to the later decades of adulthood. There is an inverse correlation between repeat length and age of onset, with longer repeats resulting in symptoms at a younger age. Expansions often increase in length in each subsequent generation, leading to a phenomenon called anticipation, where the next generation starts exhibiting symptoms at an earlier age than the previous. Reduction of repeat length has been reported but this occurs more rarely. Many individuals have repeats at an intermediate length, resulting in incomplete penetrance of the disease, but these are more likely to expand in future generations. Expansion of repeat regions can therefore appear as sporadic cases when the repeat is newly expanded, as this individual may have no other affected family members.

Repeat expansions cause disease through toxic gain of function. This gain of function can allow expanded proteins to avoid degradation, exhibit changes in expression, and influence function of other interacting genes [28,29,30]. Recently, it has been demonstrated in SCA8 and FXTAS, an X-linked ataxia, that RNA can be translated independent of a traditional ATG start site, in a process referred to as repeat-associated non-ATG translation, contributing to the harmful effects of aberrant proteins [31,32,33].

#### 3.1.2. Other Mutations in AD Ataxias

Several of the other more recently discovered dominant ataxias are caused by conventional mutations: SNVs, insertions, and deletions. Conventional mutations are much less common in AD ataxias than repeat expansions. Several mutations have only been reported in select populations or families, while others appear to exist worldwide. SCA28, for example, causes 1.5% of AD ataxia in Europeans, but has not been detected in other large populations such as Chinese [34,35]. Dominant mutations can result in disease through haploinsufficiency due to gene deletion or disruption of functionally important residues, or by dominant negative mechanisms. Although more rare than in repeat expansions, anticipation has been documented in cases of indel or SNV mutations. The mechanism behind anticipation in ataxia due to indel or SNV mutations is unknown.

The variety of mutation types present in dominant ataxias illustrates the need for careful attention to molecular assays used to screen for new mutations. *ITPR1* was initially discarded as a candidate gene but later reassessment of the same samples detected the disease-causing deletion [25,36]. The confirmation of *ITPR1* as an ataxia causing gene in humans led to the careful screening and discovery of mutations in SCA16 and SCA29 patients [26,27].

### 3.2. Autosomal Recessive

Autosomal recessive (AR) ataxias occur more frequently than AD ataxias. Known AR ataxia genes are reported in Table S2. Despite the greater frequency of AR ataxias, many of these cases go genetically undiagnosed. Often, only one individual in a family presents with recessive ataxia. These cases may appear sporadic or idiopathic, making it difficult to distinguish AR from a *de novo* AD mutation or a new expansion event. In addition, the number of genes causing AR ataxia is large, and often mutations are family-specific or private variants, which appear most frequently under conditions of a suspected founder effect or consanguineous union. Recessive ataxias, more often than dominant, have symptom onset from birth or in early childhood, but this may be due to ascertainment, and later onset recessive ataxias certainly also exist. Unlike AD, early onset AR are typically non-progressive in their symptoms, with more multisystem involvement leading to other symptoms such as intellectual disability [37,38].

The most common autosomal recessive ataxia, and the most common early onset ataxia, is Friedreich’s ataxia (FRDA). FRDA is estimated to have a prevalence of 1 in 20–50,000. In certain regions of the world, carrier rates have been estimated to be as high as 1 in 11 [39]. It is most commonly seen in individuals of European ancestry but is present worldwide. FRDA is primarily caused by a GAA intronic repeat expansion of the frataxin gene, with rare conventional mutations also reported [40,41]. The intronic expansion interferes with transcription and results in suppression of gene expression [42,43]. FRDA is a prime example where understanding the cellular pathology has guided research towards treatment, with several groups exploring methods to therapeutically increase the expression of frataxin [44], some of which are in or nearing clinical trials

Ataxia telangiectasia (A-T) is an early onset ataxia affecting 1 in 40–100,000. As mutations disrupt DNA repair, individuals with A-T are susceptible to radiation and oxidative stress. Heterozygous carriers for mutated *ATM* gene have a greater susceptibility to developing cancer. Mutations in *ATM* are highly variable, with over 600 unique variants reported.

There are several other ataxias that exhibit clinical features and molecular pathology similar to A-T. A-T like disorder is caused by mutations in *MRE11A*, another DNA repair gene. Individuals with A-T like disorder share the same neurological defects, along with oculomotor apraxia, but lack the telangiectasia and other features. Four other genes have been identified to cause ataxia with oculomotor apraxia (AOA). AOA2 is caused by mutations in *SETX* and is predicted to be responsible for 8% of non-Friedreich recessive ataxias [45]. It is prevalent among French-Canadians, but also present in other populations [45]. AOA1 is common among Japanese and Portuguese, where it was additionally characterized with features of low serum albumin and high cholesterol levels [46]. Mutations in *GRID2* and *PIK3R5*, which cause AOA and AOA3, are much less common.

### 3.3. X-Linked

In contrast to AD and AR ataxias, there are comparatively few known X-linked ataxias. The most common X-linked ataxia is fragile X-associated tremor ataxia syndrome (FXTAS). FXTAS is caused by a CGG repeat expansion in the 5' untranslated region of the *FMR1* gene [47]. This ataxia-associated expansion is often referred to as a fragile X “premutation”. The normal length of the *FMR1* repeat is less than 39 repeats, whereas 55 to 200 repeats are considered to be a premutation. Males with greater than 200 repeats have the full expansion mutation, which causes fragile X syndrome, a severe disease caused by expansion of the same repeat [48]. In the U.S., carrier rates for the *FMR1* premutation are estimated at 1 in 209 for females and 1 in 430 for males [49]. A study in a population from Quebec estimated premutation rates at 1 in 259 for females and 1 in 813 for males [50,51].

FXTAS is characterized by tremor and ataxia with late onset, usually past the fifth decade. As the gene is X-linked, males are far more commonly affected than females [52]. In female carriers, an estimated 20% experience symptoms of premature ovarian insufficiency, with onset of menopause before 40 and/or fertility issues [53]. Males with fragile X display a very different phenotype from FXTAS, with prominent intellectual disability and abnormal facial features. *FMR1* is a prime example of how subtle differences in mutations within the same gene can greatly impact the phenotype.

Other X-linked ataxias are rare, often restricted to a single family. A mutation in *ATP2B3*, also known as *PMCA3*, was recently associated with spinocerebellar ataxia in an Italian family. Researchers found a single point mutation disrupted calcium transport in the cell, resulting in a “pure cerebellar” phenotype, with congenital onset ataxia, cerebellar atrophy, hypotonia, and slow eye movements [12,54]. Although the phenotype reported is similar to that seen in other families, this is the only reported *ATP2B3* ataxia mutation to date. Sideroblastic anemia with ataxia (ASAT) is caused by mutations in *ABCB7*.

### 3.4. Mitochondrial

Mitochondrial DNA is maternally transmitted through mitochondria in the oocyte. Mutations in mitochondrial DNA genes tend to result in more multisystem diseases that can contain ataxia as a symptom. Neuropathy, ataxia, and retinitis pigmentosa (NARP) is caused by mutations in the mitochondrial DNA gene *MTATP6* [55].

Mutations in nuclear genes that function primarily in the mitochondria can also cause ataxia. Despite their association to the mitochondria, these mutations are inherited in an AR pattern. Mutations in *POLG*, which is a subunit of mitochondrial DNA polymerase, are responsible for ataxia and other multisystem features [56]. *C10orf2*, or twinkle, is necessary for proper mtDNA replication and is responsible for a variety of neurological phenotypes including infantile onset ataxia [57,58,59,60].

### 3.5. Multiple Systems Atrophy and other Multisystem Diseases that Include Ataxia

Multisystem diseases can be more difficult to diagnose due to the variability in presentation. More diverse neurological phenotypes, such as seizures and myopathy, and non-neurological symptoms such as hearing loss, cardiac problems, and diabetes can complicate these disorders. Multiple system atrophy (MSA) is a progressive neurodegenerative disorder. Individuals may initially present with Parkinsonism or ataxia, and progress to more severe cerebellar atrophy and nervous system dysfunctions. Mutations in *COQ2* shown to be responsible for MSA have been shown to be more common in the Japanese population [61]. Refsum disease can also cause cerebellar ataxia but ataxia is not present in all Refsum patients. Several members of the peroxisome biogenesis factor family are responsible for several peroxisome biogenesis disorders that can appear similar to Refsum. These diseases range in severity from resulting in early death to survival and functional ability in adulthood. The broad phenotypes displayed in these diseases can make them difficult to diagnose and classify.

## 4. Mutations in Conserved Pathways Cause Ataxia

Despite the great advances made in sequencing technology and the discovery of new genes, there is still a gap in the full understanding of the function of these gene products. Many of the functions of ataxia genes have yet to be discovered, despite overwhelming evidence that they are responsible for causing disease. Discovery of genetic pathways involved in ataxia genes is important to our understanding of disease pathogenesis, and may also impact some treatments. Expression studies and protein interaction assays focused on known ataxia genes have helped identify pathways and protein interactions [62]. Researching expression and pathways can be difficult, primarily due to the low availability of relevant tissue, as brain donation and biopsy are delicate topics for those with ataxia and their families. Knowledge of these pathways will not only be important for efforts for treatment development but aid in the discovery of new ataxia genes through the identification of common pathways and interactions. A success story for this approach is the identification of a new EA candidate gene, *UBR4*, which was selected as a candidate gene due to its role in ubiquitination and localization with another ataxia gene, *ITPR1* [63].

### 4.1. DNA Repair

The ability of a cell to repair damage to DNA is important in order to maintain proper function and avoid deleterious mutations. DNA damage can result in cell death by apoptosis or the formation of cancerous cells. Several ataxia genes have roles in DNA repair, with many involved in ataxia with oculomotor apraxia. *MRE11* acts in a complex to locate damaged DNA, where it recruits *ATM* to phosphorylate p53 and induce DNA repair [64]. In individuals with ataxia-causing *MRE11* mutations, *MRE11* fails to effectively form a complex and recruit *ATM* [65]. Mutations in *SETX*, responsible for ataxia with oculomotor apraxia, greatly decrease the ability of cells to repair double strand breaks caused by oxidative stress [66]. Single strand repair mechanisms are impaired by mutations in *TDP1* and *APTX* [67,68,69].

### 4.2. Channelopathies

Mutations in genes responsible for the transport of ions in and out of the cell result in channelopathies. Channelopathies have received the most attention as a common pathway in neurological disease, with several reviews focused on the role of channel genes in disease and neurological disorders. Defects in ion channel genes usually result in dominant negative mechanisms, as they can alter the current and exchange of ions across cell membranes, affecting cell signaling or causing intracellular accumulation. Ion voltage channels help to regulate the action potential of neurons and release neurotransmitters. EA1, EA2, and EA5 are all caused by mutations in channel genes, a potassium voltage gated channel and two calcium voltage dependent channels [24,70,71]. Two other potassium voltage gated channel genes, *KCNC3* and *KCND3*, are responsible for SCA13 and SCA19/22 [13,14,72]. Inositol 1,4,5 triphosphate binding to *ITPR1* mediates the release of calcium from intracellular stores in the endoplasmic reticulum [73,74]. Deletions in *ITPR1* are hypothesized to cause adult onset ataxia through haploinsufficiency, and mutations in conserved domains affect channel function resulting in congenital ataxia [25,26,27].

### 4.3. Ubiquitination

Ubiquitination serves multiple roles within the cell, including targeting proteins for degradation. Many ataxias result from a mutant protein escaping this degradation system. Disruption of ubiquitination systems can cause failures in many cellular processes, such as protein degradation pathways, membrane tracking, apoptosis, and immune system processes.

Several ataxia genes are in or interact with ubiquitination system proteins. Mutations in *RNF170*, an E3 ubiquitin ligase, are responsible for AD sensory ataxia [75,76]. *RNF170* was shown to associate with inositol 1,4,5-triphosphate receptors (*IP3*), while mutations in its receptor, *IP3R1* (*ITPR1*) also cause ataxia, providing a link between ubiquitination and ion channel signaling [76]. *ATXN3* is a de-ubiquitinating enzyme that interacts with parkin, an E3 ubiquitin ligase, resulting in more de-ubiquitinated parkin in the presence of *ATXN3* repeat expansion mutants [77]. Recently mutations in two different E3 ligases have been associated with ataxia and hypogonadism: *RNF216* and *STUB1* [78,79].

### 4.4. Transcription/Translation

The ability to control gene expression and protein abundance is important for proper function in the cell and organism. Failure in proper transcriptional mechanism and regulation can result in a variety of diseases including cancer, autoimmune, and neurological [80] disorders. *ATXN1* forms a complex with the transcriptional repressor capicua and may interact with the transcription factor *RORα* [29,81]. Nemo-like kinase (*NLK*) has been shown to interact with *ATXN1* transcriptional complex, and decreased expression of *NLK* positively modulates the phenotype in SCA1 models, providing another biological target for future treatments [82]. The transcription factor *RORα* exhibits decreased levels in SCA1 and SCA3, with null and mutant mice for *Rora* showing cerebellar defects and ataxia [81,83,84]. Along with DNA repair, mutations in *SETX* also interfere with transcription, highlighting interactions between senataxin and proteins involved in transcription and RNA processing [85].

## 5. Genetic Testing of Ataxias and Personalized Medicine

### 5.1. Is Genetic Testing of Ataxia Useful?

Rare forms of ataxia respond to Vitamin E or Coenzyme Q10 (AVED and SCAR9/ARCA2), but for most ataxias, only symptomatic treatment is available. Genetic testing for diseases with no treatment is controversial and in the U.S., is usually considered a personal choice, whereas developing countries do not routinely offer testing. Thus, what are the reasons to test a patient with ataxia for known genes? Indeed, some of those with ataxia have commented upon finding out that they had e.g., SCA2, so now what? There is little difference in treatment or prognosis, so why all the expense of testing?

One reason given for genetic testing is family planning. This most often arises in dominant ataxias where the disease is seen in prior generations, but can apply to recessive ataxias, especially in isolated populations where disease alleles may be at a higher frequency. Individuals who are carriers of a disease mutation may make different reproductive choices to avoid passing the disease on to the next generation. They may choose to avoid passing on genetic material by not having children, choosing adoption, or use of egg/sperm donors. Those wanting biological children may utilize pre-implantation screening with *in vitro* fertilization or termination of pregnancy after prenatal diagnosis [86]. Pre-implantation testing and testing of children have resulted in a new ethical conundrum of “genetic ignorance”, where parents may decide to remain ignorant about their own results, but wish to test offspring or embryos, possibly unnecessarily [87].

A more complex situation is that of FXTAS. This diagnosis in an older male with ataxia implies a very high risk of the more severe fragile X syndrome in any grandsons or nephews through his female relatives. Since the *FMR1* expansion is on the X chromosome, females can be asymptomatic carriers. For example, a female with a male relative with FXTAS may be a carrier, and hence be at risk of having a son with fragile X syndrome. A survey on genetic screening for *FMR1* mutations showed that while individuals are concerned about finding out they are carriers, and the emotional stress that may accompany that, many note the value in being able to make informed reproductive choices and possible benefits to other family members [88].

Genetic testing also will allow pre-symptomatic testing. One man, after finding out the genetic cause of his mother’s ataxia, decided to get tested himself before purchasing a house—he reasoned that if he had the mutation, the house should accommodate his future potential disability needs such as walker and wheel chair accessibility. Pre-symptomatic testing can result in unintended consequences for those tested, which may explain why for some neurodegenerative diseases, like Huntington’s, a minority of those at risk are tested [89,90]. There are reports of greater instances of depression in individuals with positive test results, possible stigmatization by peers or family members, or having difficulty obtaining life insurance.

Genetic testing offers a definitive diagnostic confirmation for patients. Some individuals with ataxia are first diagnosed as having amyotrophic lateral sclerosis, multiple sclerosis, MSA, or Parkinson’s. Genetic testing will become an increasingly important part of differential diagnosis in these individuals. Desire for knowledge and closure about what is causing their symptoms can be comforting to affected individuals and family members. Genetic testing also has clear clinical ramifications for prognosis. As there are clinical differences in progression rates between different forms of ataxia [21], genetic testing can help patients and their physicians understand their own prognosis. For example, SCA7 leads to severe vision problems or blindness, and SCA6 also leads to some vision problems, whereas SCA2 symptoms also include neuropathy, tremors and cramps. Rarely, Vitamin-E responsive ataxia may be confused clinically with Friedreich’s ataxia, and hence this diagnosis will open up a new treatment with large doses of vitamin E. In turn, sometimes spastic paraplegias are misdiagnosed as ataxias, and treating the spasticity aspect may bring relief [91].

### 5.2. Genetic Testing Now and in the Future Era of Cheap Sequencing

Currently, genetic testing is performed by a number of academic and commercial laboratories. For recessive ataxias, usually Friedreich’s ataxia is tested first, although depending on the symptoms, other ataxias are included. For dominant ataxias, the five most common SCAs are often tested first [92]. If these are negative, comprehensive panels for most known dominant or recessive ataxia genes, or all, are available from commercial sources [93]. Comprehensive panels are helpful when the mode of inheritance is not clear—e.g., if a parent died before symptom onset, or has some neurological symptoms that are not identical, or had been diagnosed with a different disorder.

Next generation exome sequencing has shown some success in clinical environments, demonstrating that it may be more efficient than testing for mutations in ataxia genes individually [94,95,96,97,98]. Several academic clinical laboratories offer targeted sequencing of hundreds of genes, whereas others, such as Baylor and University of Chicago, offer exome sequencing, but may specifically evaluate ataxia genes [99,100]. At what point it is best to move from sequencing genes one at a time to large panels, and when from large panels to whole exome, is currently not clear. In addition, whole exome or genome sequencing can identify mutations in genes unrelated to ataxia, such as genes associated with early onset breast cancer. This is not unique to ataxia, and how to deal with such secondary or “incidental” findings is currently actively debated by ethicists and clinicians.

In addition, there are current limitations to next generation sequencing technology. Currently large repeat expansions cannot be accurately sequenced or mapped to identify the common repeat expansion mutations. This is a limitation of the short reads captured by the sequencing technology; reads comprised of entirely repeats cannot be aligned to accurately determine placement or length. New computational methods are being developed in an attempt to tackle this problem. It is important for clinicians and genetic counselors to consider that next generation sequencing does not guarantee a diagnosis and should address this point with patients desiring sequencing.

Hence, a fully comprehensive genome analysis that covers all ataxia gene mutations is not currently available. Given the heterogeneity of ataxias, and the large number of genes still being detected each year, a comprehensive genetic test will be a challenge for researchers and clinicians.

## 6. Conclusions

The variability in phenotypic symptoms and genetic causes provide a challenge for clinicians and geneticists in studying ataxia. Advancements in sequencing technology have greatly increased our rate of discovery of new ataxia genes and ability to screen for known genes. With the price of whole exome sequencing and soon whole genome sequencing falling below $1000 a sample, it has become the cost effective approach to screen multiple genes at the same time. Continuing expression studies and investigation into the role of genes will help identify shared pathways and functions. A challenge in this movement towards next generation sequencing technology is the discovery of new repeat expansions, which are difficult to detect using this new technology. The number of known genes mutations responsible for ataxia keeps growing every year; however we still do not have well defined functions or pathways for many of these genes. With greater understanding of the pathways these genes are involved in and how each mutation causes disease, we may be able to generate more targeted and effective treatments in the future.

## Supplementary Files

Supplementary File 1
